# Is there a cardiodepressant effect of higher BMI on contractile force of skinned human fibers?

**DOI:** 10.1186/1749-8090-10-S1-A291

**Published:** 2015-12-16

**Authors:** Constanze Bening, Nicole Stumpf, Christian-Friedrich Vahl

**Affiliations:** 1 Department of Cardiothoracic and -Vascular Surgery, University Hospital Mainz, 55131, Mainz, Germany

## Background/Introduction

Body mass is reported to influence myocardial performance. Latest studies have emphasized the importance of negative inotropic Adipocyte-derived factors and assume an impact on cardiac contractile function. However the underlying mechanism remains unclear.

## Aims/Objectives

Our hypothesis was if body mass and gender impact cardiac force development on the level of the contractile apparatus.

## Method

Therefore we performed a study to examine the influence of BMI (3 groups: group I > 25, group II 25-30, group > 30) on the myocardial performance of skinned muscle fibers. Right atrial tissue preparations of 70 patients undergoing aortocoronary bypass operation (CABG, 48 patients, group a) and aortic valve replacement (AVR, 22 patients, group b) was obtained prior to cannulation and prepared for the experimental set up. The fibers were exposed to graduate increase of calcium concentration and the force values were recorded and stored by the associated computer program. The statistical analysis was performed with Pearsons's correlation (SPSS, p < 0.05 significant).

## Results

1.) BMI > 30 (group III) was associated with less force (Mean force 1.58 ± 0.1 mN, p 0.02, max force 2.24 ± 0.17 mN, p 0.02 versus group II (Mean force 1.8 ± 0.3 mN, p = 0.04, max force 2.59 ± 0.2 mN, p 0.03) and group I (Mean force 1.8 ± 0.1 mN, p = 0,03, max force 2.62 ± 0.3 mN, p 0.03). The force values between group I and II were not significantly different. Dividing the groups after surgical procedure the impact of BMI on force development in group III is even more intense in the CABG group compared to the AVR group: 2.0 ± 0.2 mN versus 2.4 ± 0.1 mN, p 0.04.

## Discussion/Conclusion

In accordance to literature, BMI > 30 is associated with reduced force capacities. Additionally the underlying cardiac disease might aggravate the impact of weight on cardiac force. Further studies are mandatory to evaluate the clinical relevance of this experimental observation and potential consequences in the therapy of obese patients.

